# *In vivo* Characterization of Amorphous Silicon Carbide As a Biomaterial for Chronic Neural Interfaces

**DOI:** 10.3389/fnins.2016.00301

**Published:** 2016-06-28

**Authors:** Gretchen L. Knaack, Daniel G. McHail, German Borda, Beomseo Koo, Nathalia Peixoto, Stuart F. Cogan, Theodore C. Dumas, Joseph J. Pancrazio

**Affiliations:** ^1^Department of Molecular Neuroscience, Krasnow Institute for Advanced Study, George Mason UniversityFairfax, VA, USA; ^2^Quantitative Scientific SolutionsArlington, VA, USA; ^3^Department of Bioengineering, George Mason UniversityFairfax, VA, USA; ^4^Electrical and Computer Engineering Department, George Mason UniversityFairfax, VA, USA; ^5^Department of Bioengineering, University of Texas at DallasRichardson, TX, USA

**Keywords:** amorphous silicon carbide, neuroprosthetic device, neural electrode, foreign body response, neuroinflammatory response, *in vivo* models

## Abstract

Implantable microelectrode arrays (MEAs) offer clinical promise for prosthetic devices by enabling restoration of communication and control of artificial limbs. While proof-of-concept recordings from MEAs have been promising, work in animal models demonstrates that the obtained signals degrade over time. Both material robustness and tissue response are acknowledged to have a role in device lifetime. Amorphous Silicon carbide (a-SiC), a robust material that is corrosion resistant, has emerged as an alternative encapsulation layer for implantable devices. We systematically examined the impact of a-SiC coating on Si probes by immunohistochemical characterization of key markers implicated in tissue-device response. After implantation, we performed device capture immunohistochemical labeling of neurons, astrocytes, and activated microglia/macrophages after 4 and 8 weeks of implantation. Neuron loss and microglia activation were similar between Si and a-SiC coated probes, while tissue implanted with a-SiC displayed a reduction in astrocytes adjacent to the probe. These results suggest that a-SiC has a similar biocompatibility profile as Si, and may be suitable for implantable MEA applications as a hermetic coating to prevent material degradation.

## Introduction

Implantable microelectrode arrays (MEAs) offer clinical potential as a neural interface for prosthetic devices by enabling restoration of communication and control of artificial limbs (Hochberg et al., 2006; Velliste et al., 2008). A variety of materials have been used to develop MEA technology including silicon, polymers, and metal (Kozai et al., 2012b; Prasad et al., 2012; Barrese et al., 2013). While proof-of-concept recordings from MEAs have been promising, work in animal models demonstrates that the obtained neural signals degrade over time (Rousche and Normann, 1998; Williams et al., 1999) and the reliability of this approach has been the subject of recent attention. Furthermore, there is significant variability in the chronic electrode performance timeline across research groups, animal models, and even within the same implant (Polikov et al., 2005).

Currently, implantable MEAs penetrate the blood brain barrier and cause shearing of vasculature, disturbances to the extracellular matrix, glia, and neurons, resulting in a neuroinflammatory response (Polikov et al., 2005; Bjornsson et al., 2006). The neuroinflammatory response is characterized by immediate activation of macrophages and microglia, which causes extension of processes toward the site (Polikov et al., 2005; Stice and Muthuswamy, 2009; Winslow and Tresco, 2010; Kozai et al., 2012a; Potter et al., 2012; Woolley et al., 2013). Astrocytes are also recruited, as indicated by upregulation of glial fibrillary acidic protein (GFAP), and extension of processes toward the implant site (Fawcett and Asher, 1999; Polikov et al., 2005; Stice and Muthuswamy, 2009; Winslow and Tresco, 2010; Bardehle et al., 2013). A significant loss of neurons proximal to devices has additionally been reported by some groups (Winslow and Tresco, 2010; Potter et al., 2012); however, the extent of this reaction is not consistent across animals and is dependent on the material, size (Stice et al., 2007), shape and insertion rate of the implant (Bjornsson et al., 2006). This neuroinflammatory response may be related to eventual device failure observed chronically (Fawcett and Asher, 1999; Winslow and Tresco, 2010), but a clear link between the tissue and device performance remains to be drawn (Polikov et al., 2005). One reason may be the variability in surgical technique, IHC protocols and data analysis between groups; standardized methodologies within the neural interface field could close this knowledge gap.

Material degradation may be another factor in device failure since several studies report structural changes of implanted MEAs including commonly used materials like silicon (Si), *in vitro* (Alexander et al., 1954) and *in vivo* (Prasad et al., 2012; Barrese et al., 2013, 2016; Kane et al., 2013). A hermetic coating of the neural interface could decrease dissolution of materials (Cogan et al., 2003; Zorman, 2009). Recently, several groups have explored the potential utility of silicon carbide (SiC) as an encapsulation layer for implantable neural devices (Cogan et al., 2003; Hsu et al., 2007; Frewin et al., 2011). SiC is a robust material that withstands high temperatures and adverse chemical conditions (Zorman, 2009), although additional material properties are dependent on the form of SiC produced. One of these types, amorphous SiC (a-SiC), has the following beneficial properties: diffusion barrier, low temperature deposition, direct binding to Si (Zorman, 2009), slow dissolution rate, and biocompatibility (Cogan et al., 2003; Iliescu et al., 2008). a-SiC has already been employed as a protective coating for orthopedic implants (Sella et al., 1993) and coronary stents (Amon et al., 1996). Moreover, efficacy in preventing both thrombogenic and inflammatory effects has been demonstrated (Hamm et al., 2003). Therefore, utilizing a-SiC as a novel material for neural interface technology could prove beneficial.

Thoroughly understanding the potential of a novel material for implantable neural devices requires systematic characterization of key markers implicated in the tissue-device response at multiple time points. Therefore, the aim of this study was to rigorously compare neuroinflammatory markers between silicon devices coated with a-SiC and uncoated silicon devices. To ensure consistency in the investigation, confounding experimental factors were minimized by standardization of device dimensions, simultaneous implantation of experimental and control probes, and counterbalancing hemisphere placement of experimental and control probes. Moreover, we performed device capture (Woolley et al., 2011) immunohistochemical labeling of neurons, astrocytes, and activated microglia/macrophages at 4 and 8 weeks; a method which assesses intact tissue slices without the removal of the implant. Neuron loss and microglia activation were similar between Si and a-SiC coated devices, while tissue implanted with a-SiC displayed a reduction in astrocytes adjacent to the neural interface. These results suggest that a-SiC has a similar biocompatibility profile as Si, and may be suitable for implantable MEA applications as a hermetic coating to prevent material degradation.

## Materials and methods

### Subjects

All animal procedures complied with the National Institutes of Health Guide for Care and Use of Laboratory Animals and were approved by the Institutional Animal Care and Use Committee (IACUC) at George Mason University. Adult female Long Evans rats (210–345 g) were chronically implanted with a non-functional neural device for either 4 weeks (*n* = 3) or 8 weeks (*n* = 5).

### Neural implants

Single shank non-functional devices were inserted into the primary motor cortex of rats. Conventional Si devices (NeuroNexus, Ann Arbor, MI) were deposited with 0.5 μm a-SiC using plasma-enhanced chemical vapor deposition (PECVD). Si devices without a-SiC were also inserted as controls. Both devices were 5 mm in length, 15 μm thick, 200 μm in width at the base and 125 μm in width at the widest part of the shank in contact with cortical tissue. The plastic holders of each device were glued together such that the shanks were parallel with each other and could be inserted simultaneously. This combined device was sterilized with ethylene oxide gas for 24 h. The methodological approach of implanting materials simultaneously into the same animal allowed us to control for variability across animals, surgery time, insertion rate, immunohistochemistry runs, and other possible confounds.

### Surgery

Rats were fully anesthetized with a 5% isoflurane/oxygen mixture at a rate of 1 L/min and maintained at 2%. Pain sensitivity was tested by paw pinches and breathing was monitored throughout surgery. Body temperature was maintained by a heating pad (Braintree Scientific, Braintree, MA). Surgical tools were sterilized with ethylene oxide gas and aseptic surgery technique was used. Each animal underwent shaving of the fur from between the eyes to behind the ears prior to placement in a stereotaxic frame (Kopf instruments, Tujunga, CA). Puralube eye ointment (PharmaDerm, Florham Park, NJ) was applied to the eyes followed immediately by a mid-scapular subcutaneous injection of dexamethasone (2 mg/kg; 2 mg/mL; Sparhawk Laboratories, Lenexa, KS) and lidocaine delivery under the scalp (2%; Clipper Distributing Company, St. Joseph, MO). The skin surface was disinfected with a 10% povidone iodide solution (Qualitest Pharmaceuticals, Inc., Huntsville, AL) before cutting a small opening with sterile scissors. The fascia was removed and the skull cleaned with three alternations of 70% ethanol and 3% hydrogen peroxide, finishing with an additional application of ethanol. Compressed air was used to dry the skull in between each application.

Drilling and probe placement were visualized through a surgical scope (Seiler Instrument and Manufacturing Company, St. Louis, MO). Six skull screws were placed in burr holes drilled with a micro drill (Stoelting, Wood Dale, IL) to anchor the skull cap. Bilateral craniotomy windows were drilled to target the primary motor cortices (from bregma: −1.5 to + 1.5 mm anterior/posterior and ±0.5 to ±2.5 mm medial/lateral). A small portion of dura was retracted, paying special attention to avoid any surface vasculature. The device tips were manually lowered to the surface of the cortex and the devices were then inserted simultaneously at a rate of 2 mm/sec with an electronic micropositioner (Kopf instruments, Tujunga, CA).

After implantation, the craniotomies were filled with silicone elastomer (Kwik-Cast, World Precision Instruments, Sarasota, FL). A layer of Loctite Prism 454 adhesive (Electron Microscopy Science, Hatfield, PA) was also applied and cured instantly with Loctite accelerant 7452 (Newark Element 14, Chicago, IL) to secure the device to the skull and screws. Dental cement (Lang Dental Manufactures, Wheeling, IL) was used to additionally secure the screws to the skull and create a robust head cap. Wound openings between the head cap and the surrounding skin were closed by attaching the skin to the dental cement using GLUture (World Precision Instruments, Sarasota, FL) and a triple-antibiotic cream was applied. To rehydrate the animals, 3 mL of 0.9% sterile saline was given subcutaneously. Ketoprofen (Fort Dodge Animal Health, Fort Dodge, Iowa) was administered subcutaneously at 5 mg/kg and continued twice daily for 3 days. Gentamicin (Clipper Distributing Company, St. Joseph, MO) was also injected subcutaneously at 8 mg/kg and continued once a day for 1 week.

### Immunohistochemistry (IHC)

Implants remained in the brain for either 4 weeks or 8 weeks. Each implant was coded as either left or right hemisphere, instead of material type, to blind the researchers and prevent experimenter bias. Rats were sacrificed by exsanguination from transcardial perfusion after anesthetizing with a 5% isoflurane/oxygen mixture (1 L/min) until no pain response was observed. Blood was cleared with phosphate buffered saline (1X PBS, Fisher Scientific, Pittsburg, PA) followed by 4% paraformaldehyde (PFA) to fix the tissue. The entire head was placed in PFA for 48 h and then stored in PBS containing 90 mg/L sodium azide (Sigma-Aldrich, St. Louis, MO) at 4°C until head caps were removed (all subsequent steps with PBS contain sodium azide).

Head caps were removed with the probes remaining in the brain for device capture IHC (Woolley et al., 2011). Briefly, dental cement and adhesive were removed by first cutting with a Dremel cutting wheel followed by drilling with a micro drill bit. The probes were separated at the base from the Si elastomer using microscissors. The elastomer was then removed using forceps and the skull was carefully detached from the brain leaving the probes intact. All of these steps were performed under a surgical scope. Brains containing the probes were then placed in fresh PBS with sodium azide and stored at 4°C until slicing.

Brains were sliced in the sagittal plane (200–250 μm) with a vibratome, such that one slice contained the entire probe. Free-floating sections were placed in a single well of a 24-well culture plate containing PBS. All slices were labeled with primary antibodies for neurons (chicken anti-NeuN, Millipore, Billerica, MA), reactive microglia/macrophages (monoclonal mouse anti-ED1 CD68, Millipore, Billerica, MA), glial fibrillary acidic protein of activated astrocytes (rabbit anti-GFAP, Dako North America Inc., Carpinteria, CA), and cellular nuclei (DAPI, Life Technologies, Grand Island, NY).

Auto fluorescence was quenched two times with 6 mg/mL sodium borohydrate (Sigma-Aldrich) for 15 min each, followed by three washes with PBS for 5 min each. Sections were incubated in blocking buffer containing 4% normal goat serum (Life Technologies) and 0.3% Triton-X 100 (Sigma-Aldrich) in PBS for 1 h followed by three rapid washes with PBS. Slices were additionally blocked with Image-iT FX (Life Technologies) for 30 min followed by three rapid washes in PBS. Slices were then incubated overnight at 4°C in primary antibody solution (CD68 1:1000, GFAP 1:500, NeuN 1:500 diluted in blocking buffer containing 4% normal goat serum and 0.1% Triton-X 100 in PBS). Following four washes with PBS for 15 min each, slices were incubated for 1 h at room temperature in fluorophore-conjugated secondary antibodies (goat anti-chicken 647, goat anti-mouse 546, goat anti-rabbit 488, 1:1000, Life Technologies) and DAPI (0.6 μm) diluted in the same blocking buffer used for the primary antibodies. To ensure homogenous penetration of all solutions slices were flipped half way through each step. Lastly, slices were washed three times in PBS for 15 min each and mounted on glass microscope slides using Fluoromount-G (Southern Biotech, Birmingham, AL).

### Imaging

All slices were imaged using a confocal microscope at 20x objective (Nikon D-eclipse C1si; Nikon Instruments, Melville, NY). Laser intensity and gain were held constant for each label across all slices. To obtain a broader field of view without losing resolution, each complete image contained 20 individual 20x images stitched together using Photoshop (Adobe Systems Inc., San Jose, CA). To control for naturally occurring variance in cell type density between cortical layers, complete images were cropped into individual layers according to Kandel et al. (2000). For some slices, the implanted devices extended past the cortex and into the white matter. These images were cropped at the end of layer six and the white matter was not analyzed.

### Data analysis

**Figure 1:**
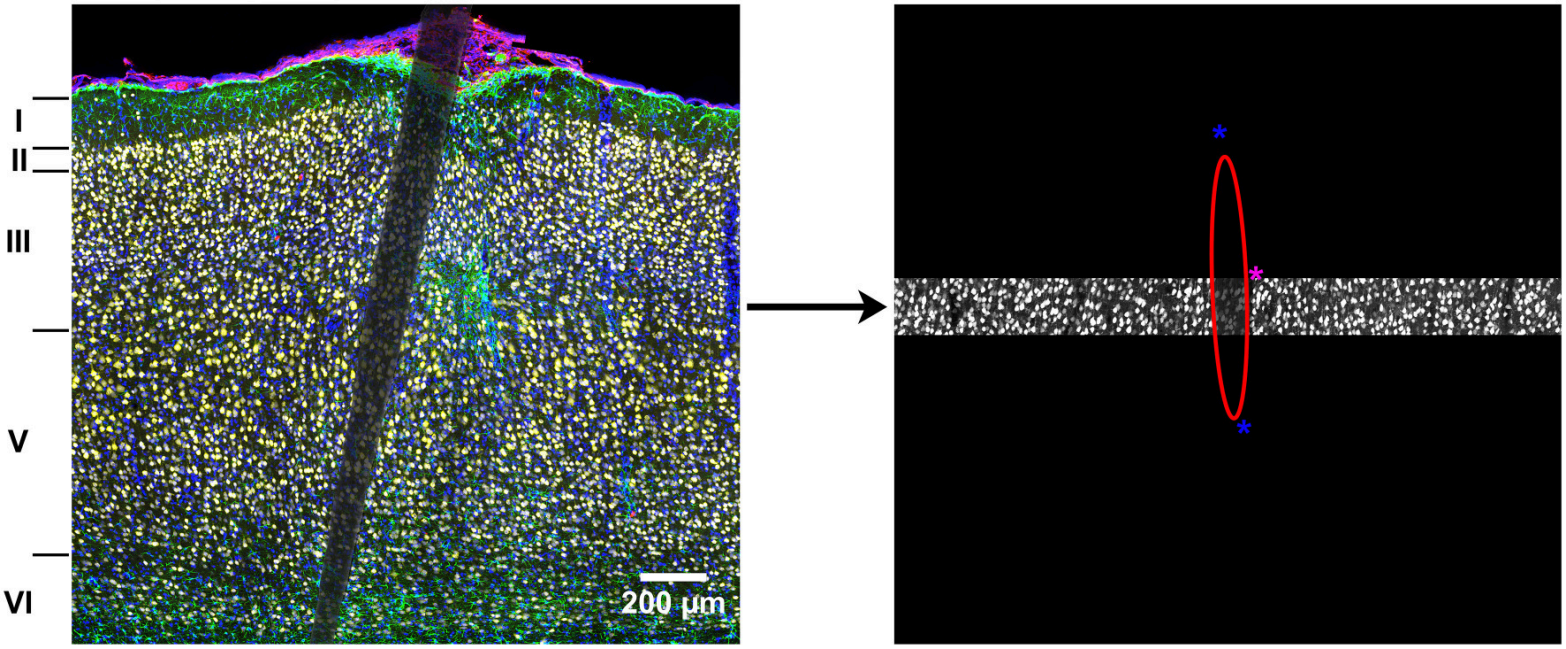
**Illustration of the analysis method**. Complete images were cropped into individual cortical layers to control for naturally occurring variance in cellular density. An ellipse was used to outline the device edges in each cropped image and fluorescence intensity was calculated every 10 μm as a function of distance from the device edge. The asterisks are part of the graphical interface and used to change the size and shape of the ellipse to fit the device edge.

Cropped images were analyzed and normalized within each cortical layer. Fluorescence intensity was measured for NeuN, GFAP, CD68, and DAPI using MINUTE (Potter et al., 2012), a custom-written MATLAB program (Math Works, Natick, MA). An ellipse was used to outline the edge of the device within each cropped image (Figure 1).

Intensity values were determined as a function of distance from the device edges every 10 μm out to a final distance of 500 μm. In order to combine fluorescence intensity, at each distance, across cortical layers, values were normalized to background within each layer as defined as the mean intensity value 400–500 μm from the probe edge, and then averaged across cortical layers to obtain a mean, normalized fluorescence intensity every 10 μm for each tissue slice. Outliers were removed if they were two standard deviations from the mean. For each label, differences in fluorescence intensity were compared using a three-way mixed ANOVA with Material (a-SiC, Si) and Time (4 weeks, 8 weeks) as between-subjects factors and Distance (0–100 μm) as a within subjects factor. A Huynh-Feldt correction was used if sphericity was violated and significance was determined as *p* < 0.05. Any significant main or interaction effects were followed up with planned contrasts due to *a priori* hypotheses where all distances were compared to baseline as defined as 100 μm from the device edge, consistent with prior work (McConnell et al., 2009; Winslow and Tresco, 2010; Azemi et al., 2011).

Additionally, area under the curve (AUC) for NeuN, GFAP, and CD68 was calculated for fluorescence intensity for 0–100 μm from the device edge, where the maximal changes were most often noted. Pearson correlations between the three targets were then conducted for 4 and 8 week time points to gain insight into the relationship between the main cell types involved in the tissue response to implanted cortical devices.

All analyses were performed using SPSS (IBM, Armonk, NY). Data are presented as mean ± standard error of the mean (SEM) unless otherwise stated.

## Results

**Figure 2:**
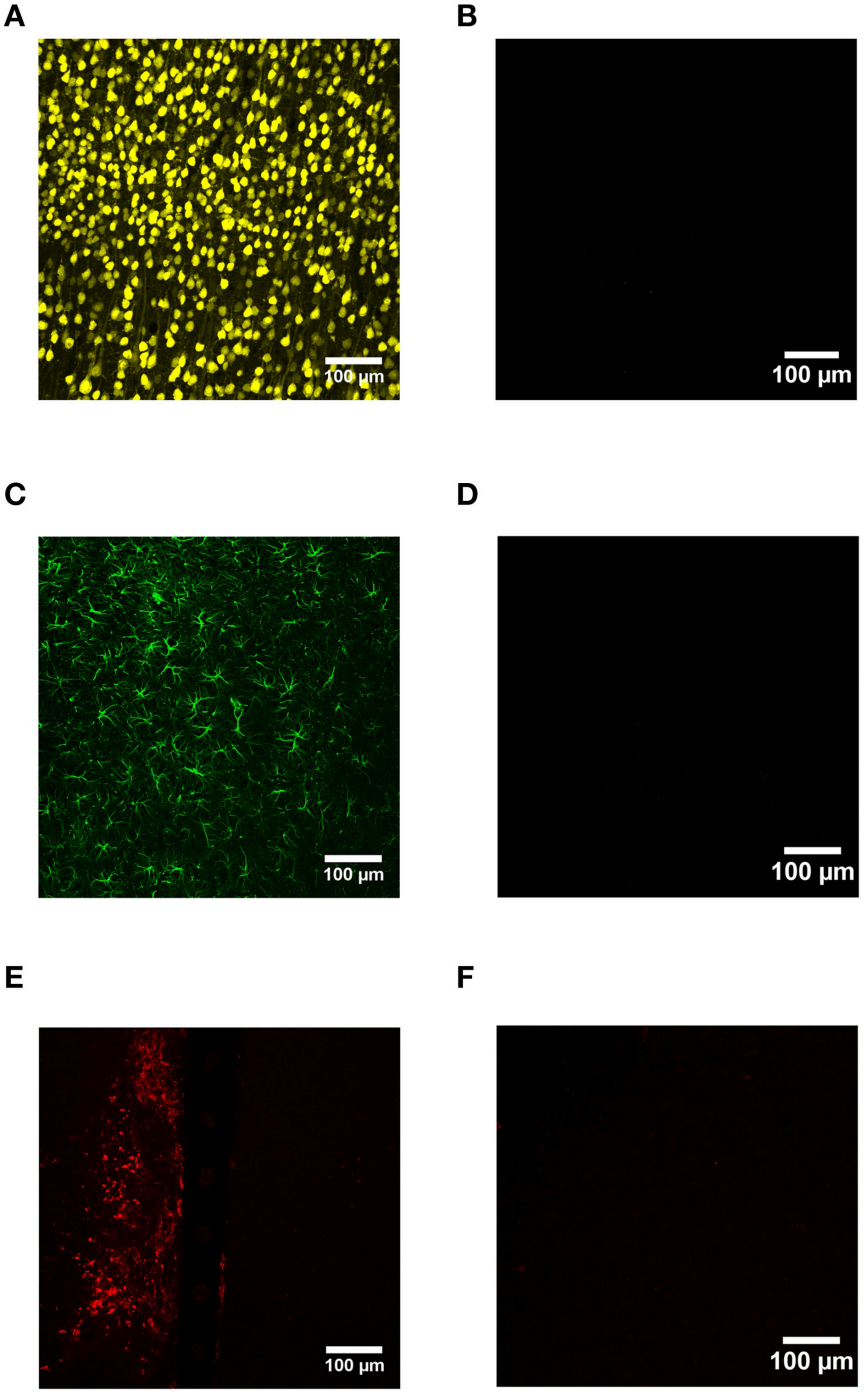
**Representative images from device capture IHC for cortical tissue compared to no primary antibody controls**. NeuN was used to label neurons **(A)**, GFAP labeled activated astrocytes **(C)**, and CD68 labeled reactive microglia/macrophages **(E)**. IHC worked with minimal non-specific binding **(B,D,F)**.

Device capture IHC was used to label neurons (NeuN), reactive astrocytes (GFAP), and activated microglia (CD68) as a function of distance from the device edge for cortical tissue implanted with Si and a-SiC coated devices for either 4 or 8 weeks. No primary antibody controls indicated that IHC worked with minimal non-specific binding (Figure 2).

### NeuN labeling of neurons

**Figure 3:**
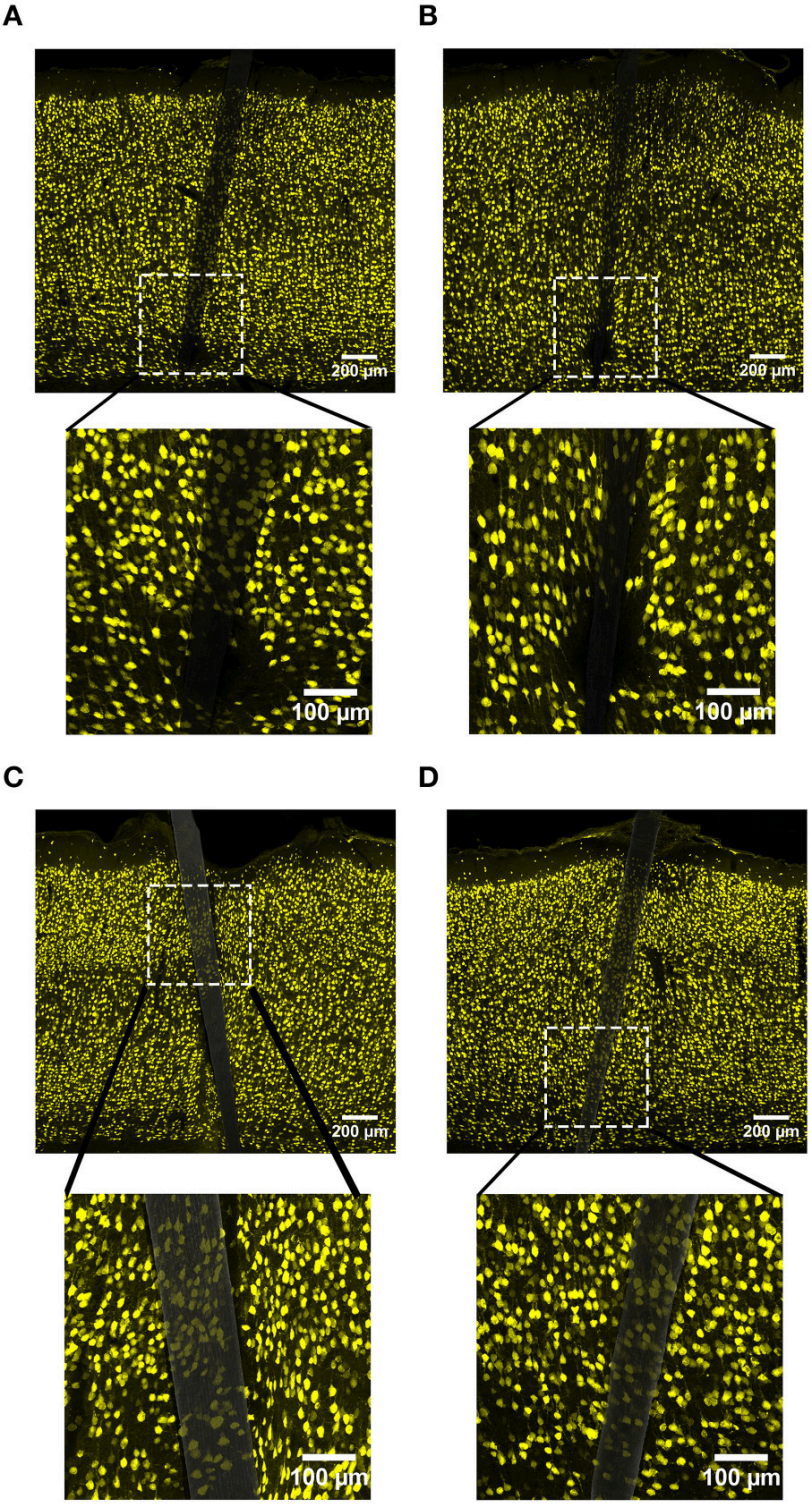
**Representative images labeling neurons (NeuN) in cortical tissue implanted with Si (A,C) and a-SiC (B,D) for 4 (A,B) and 8 weeks (C,D)**. There was less NeuN intensity near implanted devices, but an overall increase in labeling from 4 to 8 weeks.

**Figure 4:**
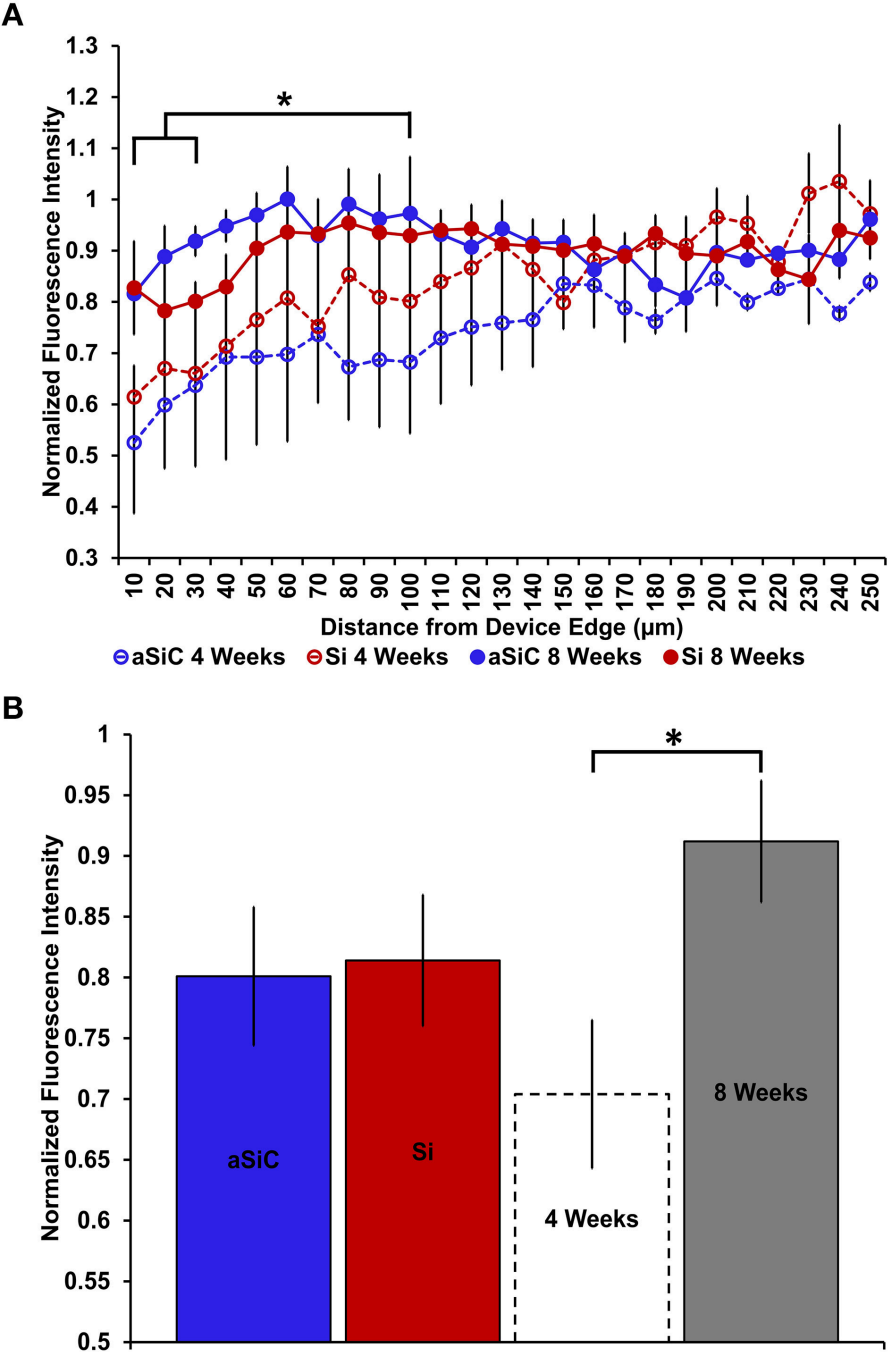
**Normalized NeuN fluorescence intensity as a function of distance from the device edge for tissue implanted with Si (red) and a-SiC (blue) probes for either 4 (dotted) or 8 weeks (solid)**. There was a significant effect of distance such that tissue 0–30 μm had significantly less NeuN labeling compared to tissue 100 μm away, regardless of material or time of implant **(A)**. There was also a main effect of time on NeuN fluorescence intensity. Tissue implanted for 8 weeks had more neurons overall compared to tissue implanted for 4 weeks regardless of material **(B)**. Data are mean ± SEM, ^*^denotes *p* < 0.05.

NeuN labeling displayed a decrease in intensity near implanted devices; however, there appeared to be a recovery in NeuN intensity with time of implant (Figure 3), regardless of whether or not the probe was coated with a-SiC. A three-way mixed ANOVA of NeuN revealed that there was a significant main effect of distance [*F*_(5.077, 55.85)_ = 5.67, *p* < 0.001], indicating that there was less NeuN intensity proximal to devices and this observation was independent of implanted material or time point. Planned contrasts determined that the reduction in NeuN labeling was located 0–30 μm from the device edge compared to 100 μm away (Figure 4A). Mean NeuN intensity from 0 to 30 μm was 86% of the intensity measured at distal locations (100 μm) from the device edge for both experimental and control probes. There was also a significant main effect of time on NeuN fluorescence intensity [*F*_(1, 11)_ = 6.85, *p* < 0.05]. Tissue implanted for 8 weeks had 30% more NeuN labeling compared to tissue implanted for 4 weeks regardless of material or distance from the device (Figure 4B). There was no main effect of material or interaction effects between material, distance, or time on NeuN fluorescence intensity. These data suggest that a-SiC coated devices did not differ from uncoated Si devices with respect to the density of proximal neurons.

### GFAP labeling of astrocytes

**Figure 5:**
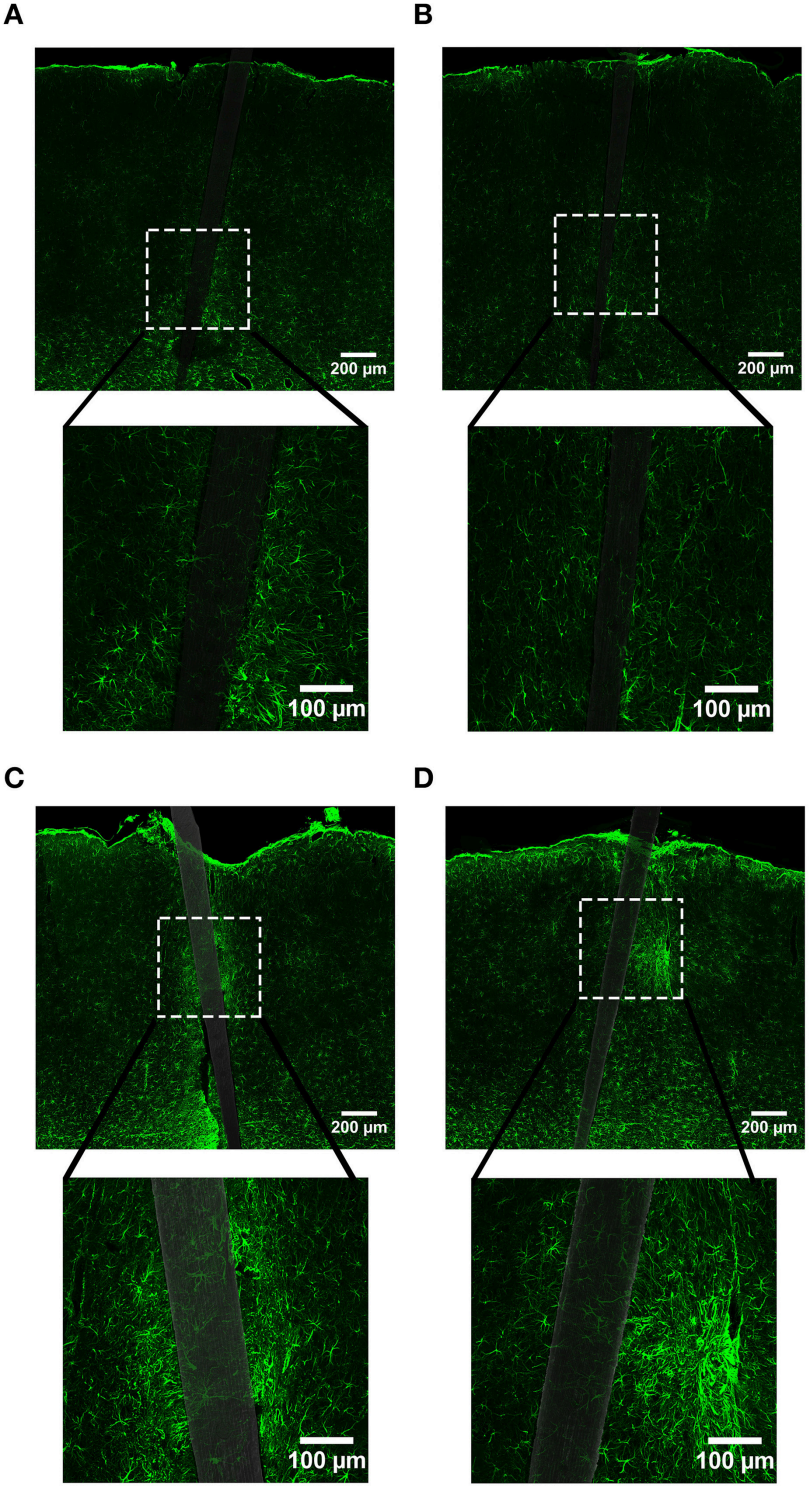
**Representative images labeling astrocytes (GFAP) in cortical tissue implanted with Si (A,C) and a-SiC (B,D) for 4 (A,B) and 8 weeks (C,D)**. There was a visibly apparent increase in GFAP intensity closer to implanted devices and this enhancement was larger for tissue implanted with Si compared to tissue implanted with a-SiC. Additionally, there was an overall increase in GFAP from 4 to 8 weeks, regardless of whether the probe was coated with a-SiC.

**Figure 6:**
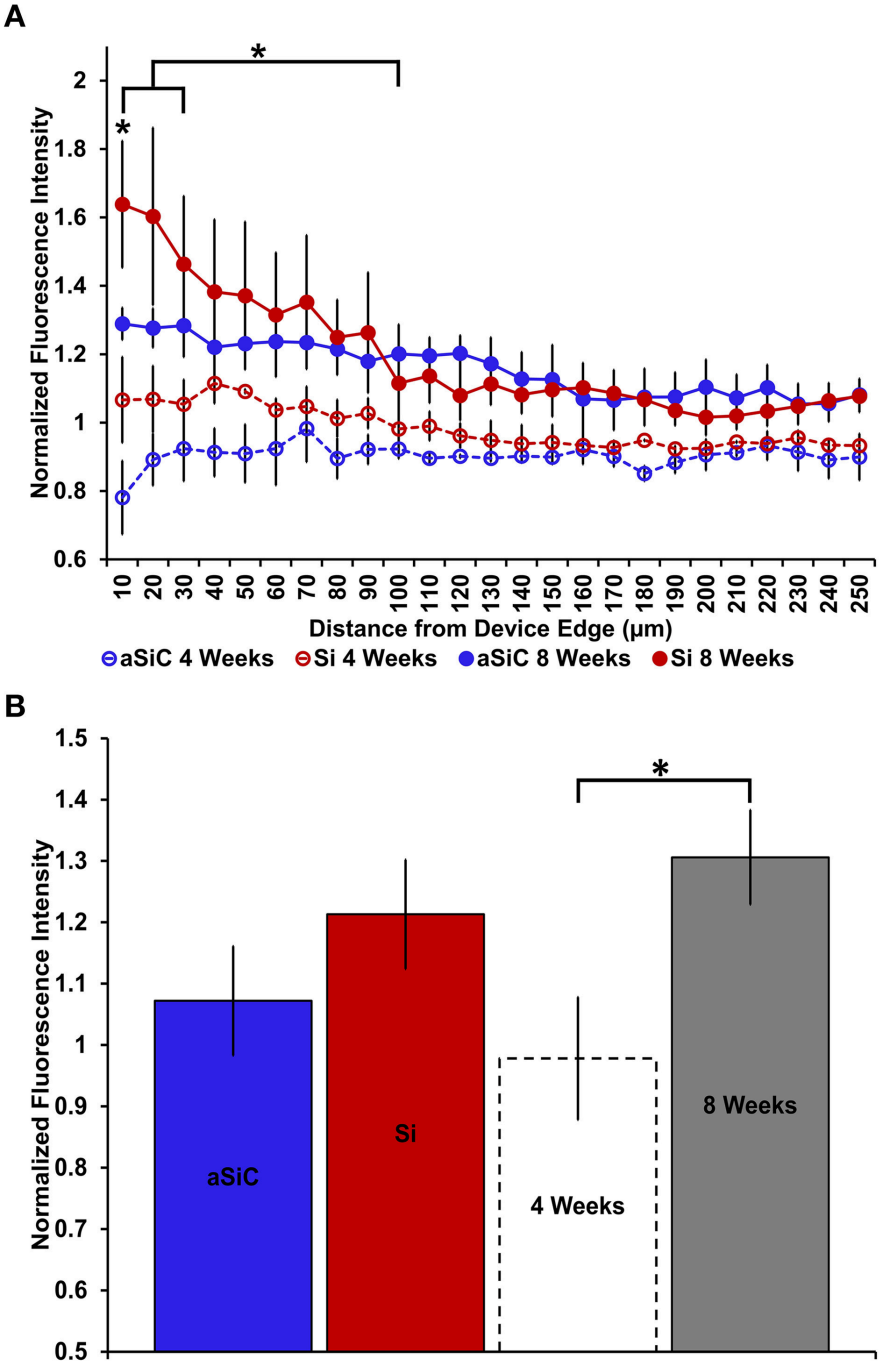
**Normalized GFAP fluorescence intensity as a function of distance from the device edge for tissue implanted with Si (red) and a-SiC (blue) probes for either 4 (dotted) or 8 weeks (solid)**. There was a significant effect of distance such that tissue 0–30 μm from the device edge had significantly more GFAP labeling compared to tissue 100 μm away, regardless of material or time of implant **(A)**. There was an interaction effect between distance and material where tissue implanted with Si devices had more GFAP labeling within 0–10 μm than tissue implanted with a-SiC devices **(A)**. There was also an interaction effect between distance and time of implant such that tissue implanted for 8 weeks had more GFAP labeling within 0–10 μm than tissue implanted for 4 weeks **(A)**. There was a significant effect of time of implant where tissue implanted for 8 weeks had higher overall GFAP intensity than tissue implanted for 4 weeks regardless of material **(B)**. Data are mean ± SEM, ^*^denotes *p* < 0.05.

In contrast to what we observed with NeuN, an increase in GFAP labeling near each device type was visually apparent, but the level of increase appeared to be material and time dependent (Figure 5). A three-way mixed ANOVA of GFAP determined that there was a significant main effect of distance [*F*_(4.03, 48.36)_ = 3.69, *p* < 0.01], such that there was more intense GFAP signal near the device edge regardless of implanted material or time of implant. Planned contrasts indicated that the enhanced GFAP labeling was specifically 0–30 μm from the device, with a mean intensity value 13% higher overall, compared to 100 μm away (Figure 6A). There was an interaction effect between distance and material [*F*_(4.03, 48.36)_ = 2.71, *p* < 0.05] where the intensity of GFAP labeling proximal to Si devices was 31% greater, compared to GFAP labeling proximal to a-SiC devices. Planned contrasts specified that the difference detected between materials was located within 0–10 μm from the device edge (Figure 6A). There was also an interaction effect between distance and time of implant [*F*_(4.03, 48.36)_ = 2.56, *p* < 0.05], indicating that the intensity of GFAP labeling decreased with distance from the device differently depending on the time of implant. Planned contrasts determined that tissue implanted for 8 weeks had 58% more GFAP labeling within 0–10 μm than tissue implanted for 4 weeks (Figure 6A) and this effect was independent of material type. Lastly, there was a main effect of time on GFAP intensity [*F*_(1, 12)_ = 6.71, *p* < 0.05], such that tissue implanted for 8 weeks had 34% more labeling in general than tissue implanted for 4 weeks, regardless of material or distance from the device (Figure 6B). There was no main effect of material on GFAP intensity. These data jointly suggest that although increased astrocyte recruitment was observed proximal to all devices, there appears to be a reduced astrocyte response directly adjacent to devices coated with a-SiC.

### CD68 labeling of activated microglia/macrophages

**Figure 7:**
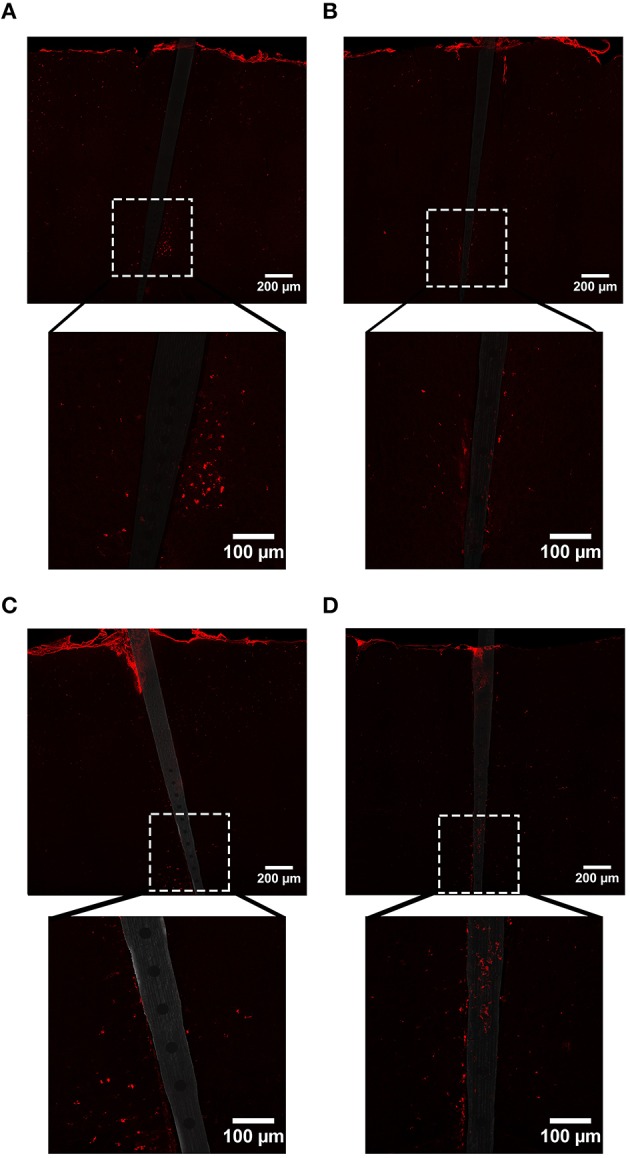
**Representative images labeling reactive microglia/macrophages (CD68) in cortical tissue implanted with Si (A,C) and a-SiC (B,D) for 4 (A,B) and 8 weeks (C,D)**. There was an increase in CD68 intensity proximal to implanted devices and this magnification was larger for tissue implanted for 8 weeks compared to tissue implanted for 4 weeks, regardless of probe material.

**Figure 8:**
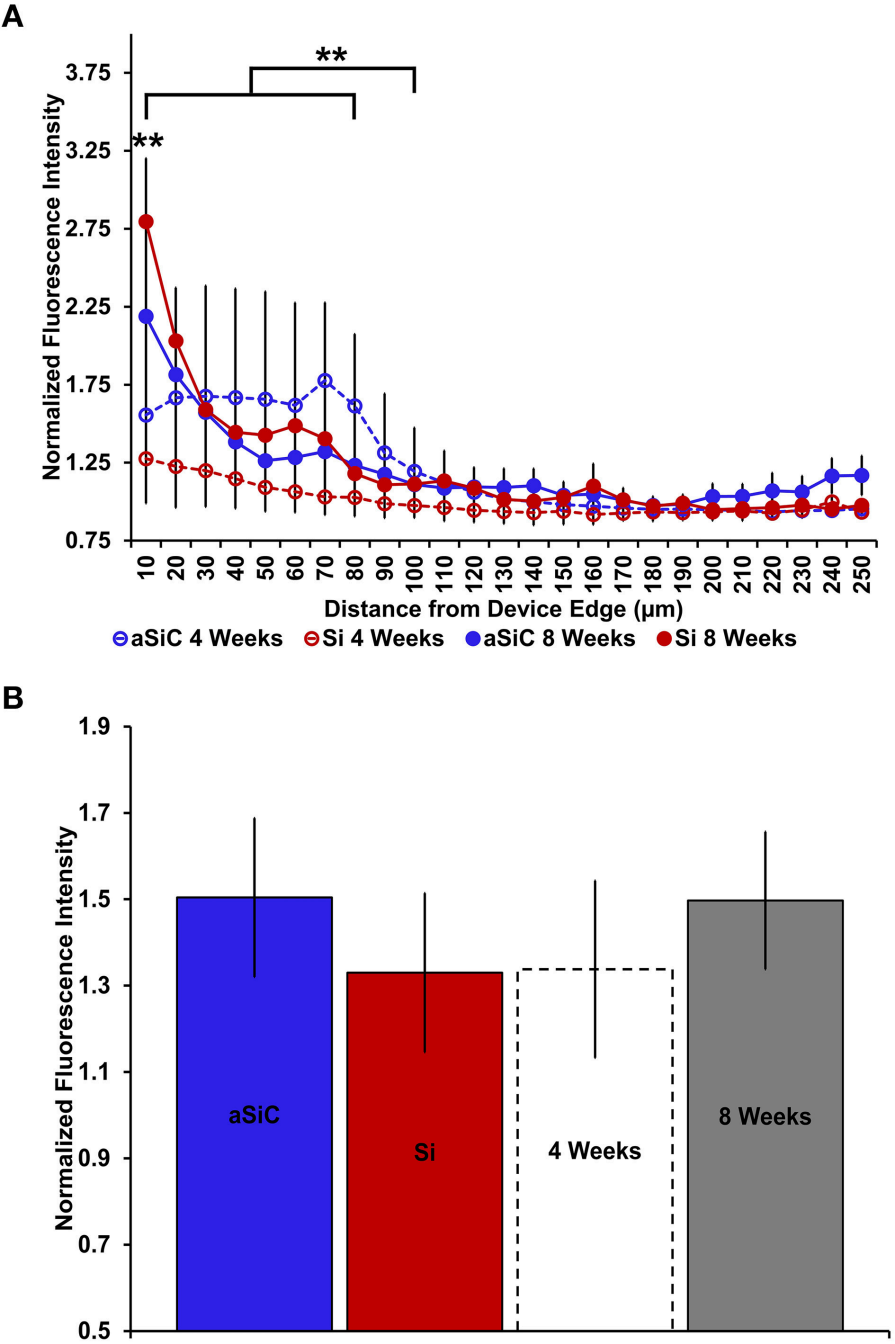
**Normalized CD68 fluorescence intensity as a function of distance from the device edge for tissue implanted with Si (red) and a-SiC (blue) probes for either 4 (dotted) or 8 weeks (solid)**. There was a significant effect of distance such that tissue 0–80 μm from the probe edge had more CD68 labeling compared to tissue 100 μm away, regardless of material or time of implant **(A)**. There was also a significant interaction effect between distance and time of implant where tissue implanted for 8 weeks had higher CD68 intensity within 0–10 μm than tissue implanted for 4 weeks **(A)**. There was no overall effect of material **(B)**. Data are mean ± SEM, ^**^denotes *p* < 0.01.

Similar to our GFAP findings, enhanced CD68 labeling was observed near both uncoated and a-SiC coated implanted devices, but the level of increase was dependent on the duration of implantation (Figure 7). A three-way mixed ANOVA of CD68 determined that there was a significant main effect of distance [*F*_(2.66, 31.97)_ = 14.22, *p* < 0.001], indicating that CD68 fluorescence intensity was greater near the probe edge regardless of material or duration of implant. Planned contrasts denoted that the increase in CD68 signal spanned from 0 to 80 μm from the probe, with a 36% higher mean intensity value compared to 100 μm away (Figure 8A), and this effect was independent of material. There was also an interaction effect between distance and time of implant [*F*_(2.66, 31.97)_ = 5.05, *p* < 0.01]. Planned contrasts revealed that the difference in CD68 intensity between durations of implant was confined to 0–10 μm from the probe, where tissue implanted for 8 weeks had 76% more fluorescence than tissue implanted for 4 weeks (Figure 8A), regardless of device coating. There was no main effect of material or time on CD68 fluorescence intensity (Figure 8B) or an interaction effect between material and distance. These data suggest that the profile of activated microglia/macrophages is similar between uncoated Si and a-SiC coated devices. Interestingly, we detected a significant negative correlation between CD68 and NeuN at the 4 week time point [*r* = −0.92, *p*(one-tailed) < 0.01], a relationship which was not apparent at the 8 week time point. Likewise, there was a significant negative correlation between CD68 and GFAP at 4 weeks [*r* = −0.79, *p*(one-tailed) < 0.05] which also dissipated at 8 weeks. These data jointly suggest that as the density of reactive microglia/macrophages increases, the density of both neurons and astrocytes decreases.

### DAPI labeling of cellular nuclei

**Figure 9:**
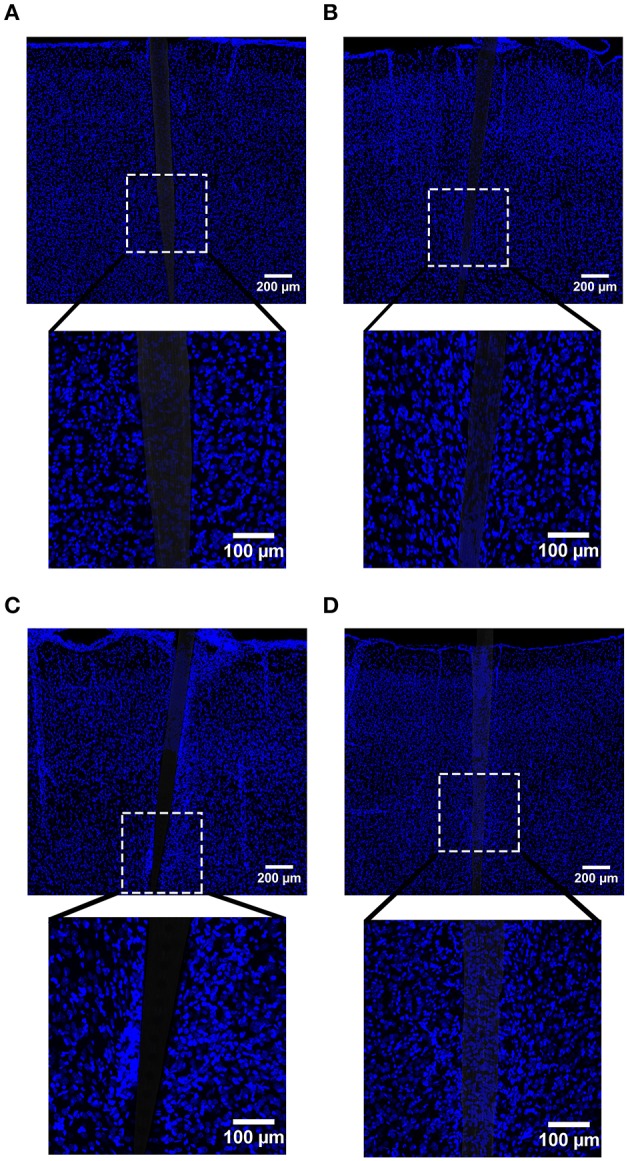
**Representative images labeling cellular nuclei (DAPI) in cortical tissue implanted with Si (A,C) and a-SiC (B,D) for 4 (A,B) and 8 weeks (C,D)**. There was an increase in DAPI intensity near implanted devices and this amplification was larger for tissue implanted for 8 weeks compared to tissue implanted for 4 weeks, regardless of whether the probe was coated with a-SiC.

**Figure 10:**
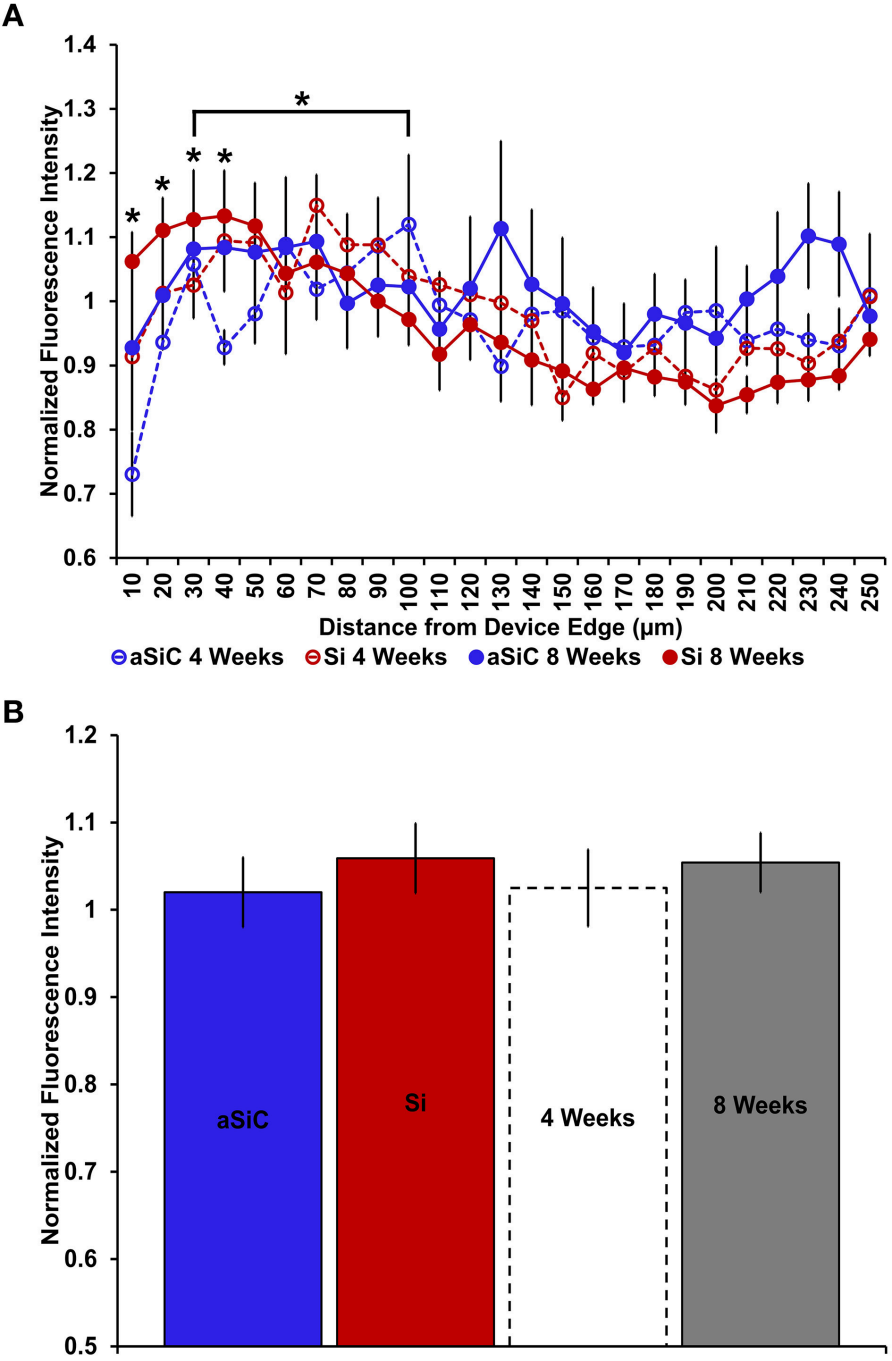
**Normalized DAPI fluorescence intensity as a function of distance from the device edge for tissue implanted with Si (red) and a-SiC (blue) probes for either 4 (dotted) or 8 weeks (solid)**. There was a significant effect of distance such that tissue 30 μm from device edge had more DAPI labeling compared to 100 μm away, regardless of material or time of implant **(A)**. There was also a significant interaction effect between distance and time of implant where tissue implanted for 8 weeks had higher DAPI intensity within 0–40 μm than tissue implanted for 4 weeks **(A)**. There was no overall effect of material **(B)**. Data are mean ± SEM, ^*^denotes *p* < 0.05.

**Figure 11:**
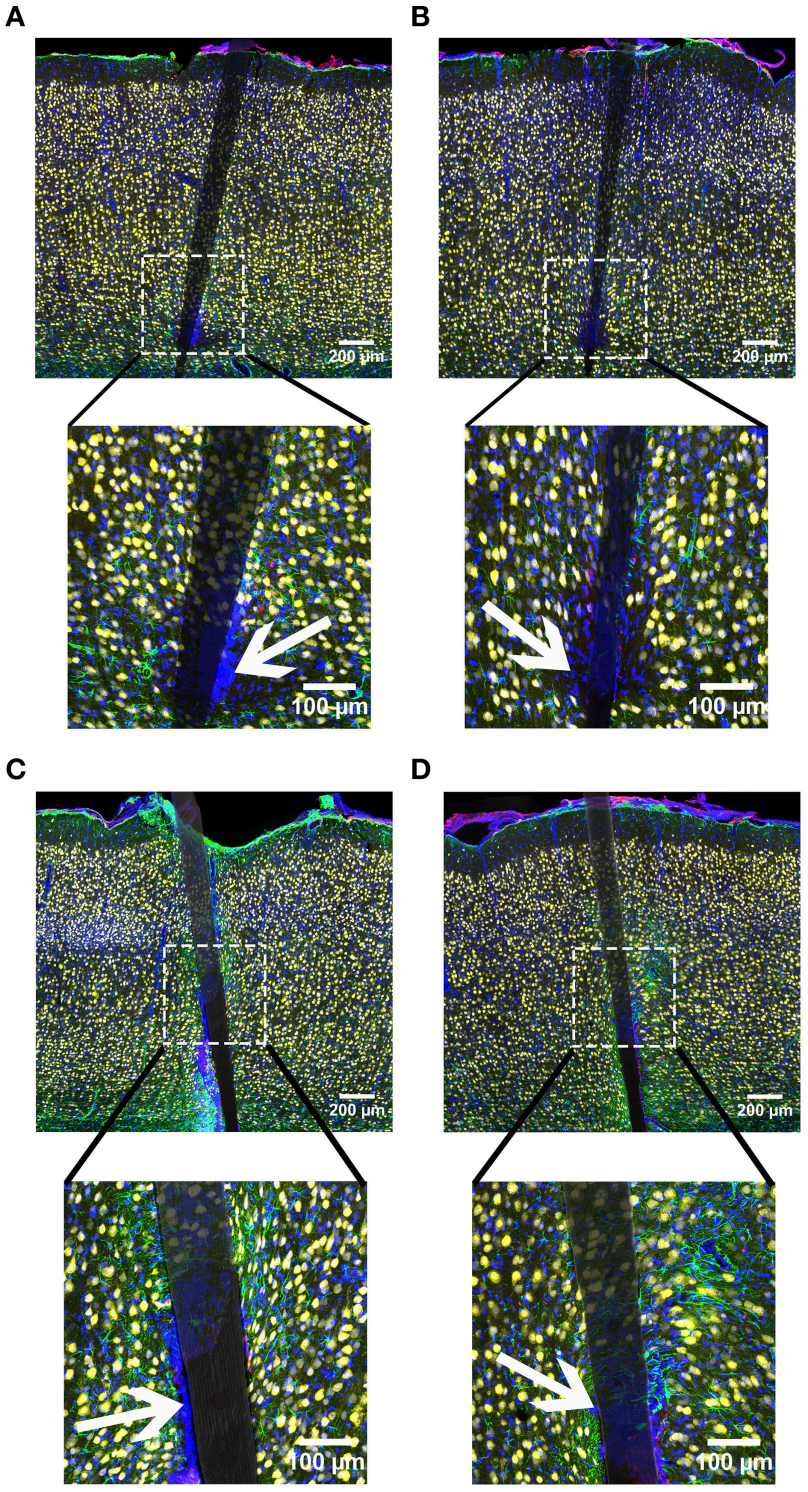
**Representative images from device capture IHC for cortical tissue implanted with Si (A,C) and a-SiC (B,D) for 4 (A,B) and 8 weeks (C,D)**. Neurons (NeuN) are yellow, activated astrocytes (GFAP) are green, reactive microglia/macrophages (CD68) are red, and cellular nuclei (DAPI) are blue. There was an increase of all labels for tissue implanted for 8 weeks compared to tissue implanted for 4 weeks. Interestingly, there were DAPI labeled cells that did not co-localize with any other label. Representative cells indicated by white arrows. These cells were visible in all groups and were always located next to the device.

In line with our GFAP and CD68 results, DAPI labeling exhibited an augmented signal near uncoated Si and a-SiC coated devices, the extent of which was implant duration dependent (Figure 9). A three-way mixed ANOVA of DAPI intensity denoted that there was a significant main effect of distance [*F*_(6.87, 82.38)_ = 3.08, *p* < 0.05]. Planned contrasts indicated that the increased DAPI labeling was specifically 30 μm from the device, compared to 100 μm (Figure 10A), regardless of material. There was also an interaction effect between distance and time of implant [*F*_(6.87, 82.38)_ = 2.43, *p* < 0.05]. Planned contrasts indicated that tissue implanted for 8 weeks had 11% more DAPI labeling within 0–40 μm than tissue implanted for 4 weeks (Figure 10A) and this finding was independent of coating. There was no main effect of material or time on DAPI fluorescence intensity (Figure 10B) or an interaction effect between material and distance. Interestingly, there were DAPI labeled cells that did not co-localize with any other label. These cells were visibly apparent in all groups and were always located next to the device (Figure 11). These data indicate that the overall cell density did not differ between uncoated Si probes and a-SiC coated probes, but also suggest that there may be another cell type of interest.

## Discussion

Using fluorescence based immunohistochemistry, we have systematically examined the neuroinflammatory response between uncoated and a-SiC-coated implantable devices. Our results indicate that a-SiC coated devices exhibit a similar overall profile as control devices, may offer a reduction in the inflammatory response of astrocytes within 10 μm of the device edge, and should be further investigated as a possible hermetic coating for neural implants.

To assess the effects of chronic implants on neuronal density, NeuN intensity was calculated as a function of distance from the edge of the implant. Similar NeuN intensity was observed between tissue implanted with a-SiC coated devices and tissue implanted with Si. This finding is in line with another report of comparable neuronal density between a-SiC coated devices and controls implanted into the parietal cortex of rabbits (Cogan et al., 2003). Overall, there was less neuronal density within the first 30 μm of the implant, but NeuN labeling returned to baseline at further distances. The decrease in labeling proximal to the devices is consistent with the literature (Biran et al., 2005; McConnell et al., 2009; Winslow and Tresco, 2010; Potter et al., 2012) and may result from the formation of the glial scar encapsulating the device, from neuronal death caused during insertion, or from unhealthy neurons not expressing NeuN. This finding occurred for both materials and time points and parallels previous reports of unaffected neuronal density 100 μm from a-SiC coated implants in Cogan et al. (2003). Interestingly, there was an overall increase in neuronal density for tissue implanted for 8 weeks compared to tissue implanted for 4 weeks and this result was independent of material. The detection of NeuN recover over time is consistent with previous findings (Nguyen et al., 2014), but does differ from one report (Potter et al., 2012); however, there were methodological differences in the preparation of the tissue, which may create differences in measurements proximal to the device. Characteristic differences have been noted for acute and chronic stages of the neuroinflammatory response (Turner et al., 1999; Szarowski et al., 2003; McConnell et al., 2009; Nguyen et al., 2014) and these data suggest that NeuN recovery is one aspect. Since the loss of NeuN was more significant at 4 weeks, astrocyte recruitment was higher at 8 weeks, and increased NeuN was also detected at 8 weeks, it is unlikely that the neuronal loss proximal to implanted devices is directly caused by the formation of the glial scar and is more likely due to insertion or unhealthy neurons not expressing NeuN.

Markers of the neuroinflammatory response were also labeled and quantified. In contrast with NeuN, tissue implanted with a-SiC coated devices had less GFAP intensity within 0–10 μm, than tissue implanted with Si devices, suggesting that a-SiC may attenuate astrocyte reactivity in tissue closest to the device. For all groups, there was more GFAP labeling proximal to devices compared to further distances, which is consistent with the glial encapsulation as previously shown (Turner et al., 1999; Polikov et al., 2005; McConnell et al., 2009; Winslow and Tresco, 2010; Potter et al., 2012; Nguyen et al., 2014). Additionally, there was increased GFAP labeling that extended more distal in tissue implanted for 8 weeks compared to tissue implanted for 4 weeks. Jointly, these data suggest that the astrocyte reaction is more intense adjacent to the device and stretches further in tissue implanted for 8 weeks. This is in line with previous studies that also found increased GFAP after 8 weeks of implantation and characterized it as a more compact sheath (Turner et al., 1999; Szarowski et al., 2003; McConnell et al., 2009; Nguyen et al., 2014). In contrast, one group detected less GFAP at 8 weeks (Potter et al., 2012) and another group detected no difference in GFAP between 4 weeks and later time points (Winslow and Tresco, 2010). These discrepancies may again be attributed to methodological differences including removal of the device from the tissue.

Similar to NeuN, CD68 labeling was consistent between tissue implanted with a-SiC coated devices and control tissue and suggests a comparable reactive microglia/macrophage response. In line with GFAP results, there was enhanced CD68 labeling proximal to all devices and the level of increase differed between lengths of implant. Increased reactive microglia/macrophages near devices is supported by previous studies (Biran et al., 2005; Polikov et al., 2005; McConnell et al., 2009; Winslow and Tresco, 2010; Woolley et al., 2011; Nguyen et al., 2014) and results from the inflammatory response. We additionally detected enhanced CD68 labeling at 8 weeks compared to 4 weeks, which is consistent with one other group (McConnell et al., 2009). In contrast, a decrease from 4 to 8 weeks (Potter et al., 2012) and no statistical differences between time points (Winslow and Tresco, 2010) has also been reported. As with the other labels, variance across the literature may result from methodological differences.

There was also an increase in DAPI labeling near devices and this enlarged signal was dependent on the time of implant, which parallels GFAP and CD68, but contrasts with NeuN. Interestingly, when images of all cellular markers were examined, it was noted that there were numerous DAPI labeled cells that did not co-localize with NeuN, GFAP, or CD68. These cells were visibly apparent in all groups and were always located next to the device. In these regions, there was a much larger loss of NeuN and more intense CD68 labeling. Since we only labeled reactive microglia/macrophages it is possible that these cells could be resting/ramified microglia (Polikov et al., 2005) or M2-type macrophages (Kigerl et al., 2009). However, the pattern with CD68 and NeuN implies a proinflammatory cell type. A similar observation was reported in Woolley et al. (2013) after 4 weeks of implantation and these cells were presumed to be meningeal fibroblasts due to vimentin labeling. Other groups have also detected connective tissue and the presence of the extracellular matrix in the glial scar surrounding transcranial devices (Stensaas and Stensaas, 1976; Liu et al., 1999; Kim et al., 2004). However, this phenomenon was only observed in the superficial layers of the cortex (Woolley et al., 2013), but in the current study the unidentified cells were located in all cortical layers. Numerous other cell types have been linked to the neuroinflammatory response and could be possibilities including monocytes, mast cells, pericytes, T-cells, B-cells, and lymphocytes (Ransohoff and Engelhardt, 2012; Skaper et al., 2012; Jansson et al., 2014). Future research should investigate the identity of this cell type(s) and characterize its role in the neuroinflammatory response to cortical implants.

Since tissue implanted with a-SiC coated devices did not statistically differ in NeuN, CD68, and DAPI labeling from tissue implanted with standard Si devices, a-SiC is no less biocompatible than Si for *in vivo* use with chronic neural implants. This is in line with other *in vivo* studies which detected no significant difference between a-SiC coated samples and controls for the neuroinflammatory response in the parietal cortex and the subcutaneous inflammatory response (Cogan et al., 2003). a-SiC coatings have also been evaluated as biocompatible for use with coronary stents (Bertrand et al., 1998) and orthopedic implants (Sella et al., 1993). Furthermore, we present data showing that the addition of a-SiC significantly reduced GFAP labeling adjacent to the devices, which suggests that a-SiC may reduce the astrocyte component of the neuroinflammatory response. The neuroinflammatory data presented here, paired with the known material properties of a-SiC, jointly suggest that a-SiC should be further investigated as a hermetic coating to prevent material degradation of neural implants.

Given the inconsistencies in the literature regarding the characterization of the neuroinflammatory response to implantable devices over time, the neural interface field would benefit from standardization of experimental methods so that a better understanding of the biology can permit optimization of the technology. Removal of the device prior to assessment, likely eliminates the tissue most influenced by the implant. Most of the significant cell changes detected in this study were only within 0–80 μm from the device edge and may not have been detected if the devices were removed. Although, the device capture technique has been implemented before, few groups have adopted this technique and the current study expanded this method by comparing between structurally defined cortical layers and not arbitrary cortical depth. This permitted for the control of naturally occurring differences in cell density and only compare changes induced by the implanted devices. Furthermore, by leaving the device intact in the tissue, the current work was able to identify an unknown cell population located on and directly next to the devices that seems to be specifically related to the biological response of the host to biomaterials. Lastly, these experimental methodologies facilitated the detection of statistically significant relationships between neurons, astrocytes, and activated microglia/macrophages in response to the devices and evaluate how these associations changed with duration of implant, relationships which have not been reported previously.

## Author contributions

Conceived and designed the experiments: GK, NP, SC, TD, JP. Performed the experiments: GK, DM, TD. Analyzed the data: GK, DM, GB, BK. Contributed to drafting and reviewing the manuscript: GK, NP, SC, TD, JP.

### Conflict of interest statement

The authors declare that the research was conducted in the absence of any commercial or financial relationships that could be construed as a potential conflict of interest.
